# Prognostic impact of a past or synchronous second cancer in diffuse large B cell lymphoma

**DOI:** 10.1038/s41408-017-0043-6

**Published:** 2018-01-25

**Authors:** Kazuna Tanba, Yoshiaki Chinen, Hitoji Uchiyama, Nobuhiko Uoshima, Kazuho Shimura, Shinichi Fuchida, Miki Kiyota, Mitsushige Nakao, Yuji Shimura, Tsutomu Kobayashi, Shigeo Horiike, Katsuya Wada, Chihiro Shimazaki, Hiroto Kaneko, Yutaka Kobayashi, Masafumi Taniwaki, Junya Kuroda

**Affiliations:** 1 Division of Hematology and Oncology, Department of Medicine, Kyoto Prefectural University of Medicine, Kyoto, Japan; 2 Kyoto Clinical Hematology Study Group, Kyoto, Japan; 3 Department of Hematology, Japanese Red Cross Kyoto Daiichi Hospital, Kyoto, Japan; 4 Department of Hematology, Japanese Red Cross Kyoto Daini Hospital, Kyoto, Japan; 5 Department of Hematology, Aiseikai Yamashina Hospital, Kyoto, Japan; 6 Department of Hematology, Japan Community Health Care Organization Kyoto Kuramaguchi Medical Center, Kyoto, Japan; 7 Department of Hematology, Matsushita Memorial Hospital, Osaka, Japan; 8 Department of Internal Medicine, Otsu Municipal Hospital, Shiga, Japan

Therapeutic improvements for cancers in general have resulted in a growing population of cancer survivors at risk of developing secondary primary malignancies (SPMs) due to a variety of biological factors such as cancer predisposition syndromes, environmental factors, immune impairments, and late effects of genotoxic therapies. Patients with a history of other primary malignancies have the possibility of dose-reduced treatment because of the chronic health problems induced by previous cancer treatment or previous cancer damage^1–5^. In addition, when analyzing the prognostic factors, death due to other cancers before disease progression is considered a competing risk^6^.

This could also be the case with diffuse large B-cell lymphoma (DLBCL), the most prevalent subtype of non-Hodgkin lymphoma^7^. While the recent treatment progress has markedly improved overall long-term outcomes for DLBCL by the advent of the immunochemotherapy containing rituximab^1^, the clinical and prognostic impacts of a history of past cancer or co-existence of another synchronous cancer in “secondary” or “concomitant” DLBCL has been rarely investigated in the recent era. To answer this question, we performed a retrospective analysis of 809 DLBCL patients who were diagnosed histologically and treated between January 2006 and February 2016 at institutes in the Kyoto Clinical Hematology Study Group (KOTOSG). The criteria for multiple primary malignancy (MPM) used were proposed by Warren and Gates: i) tumors have definite features of malignancy, ii) tumors are separate and distinct from the index tumor, and iii) the possibility of a tumor being a metastasis of the index tumor is ruled out^8^. Based on the Surveillance Epidemiology and End Results Program definition for the chronicity of MPM^9^, the term “synchronous” refers to a condition where more than two malignancies, including DLBCL, are detected within two months, while “past” indicates a metachronous condition where the tumors are detected more than two months apart. This study was approved by the Institutional Review Boards.

The 809 DLBCL patients were classified into three groups: those with past cancer (i.e., patients with precedent cancer(s) that occurred more than two months before the diagnosis of DLBCL); those with synchronous cancer (i.e., patients diagnosed with DLBCL and other malignancies within two months of each other); and patients without another cancer. Among them, 123 (15.2%) were defined as having MPM, including 94 with past cancer and 29 with synchronous cancer (Supplementary Table S1). DLBCL patients with MPM were significantly older than those without MPM (75 vs. 70 years old; *P* < 0.001). There was no significant difference in disease stage or international prognostic index (IPI)-defined disease risk between patients with and without MPM. Although small adjustments of the therapeutic regimen were allowed at the doctor’s discretion, more than 85% of patients received a rituximab plus CHOP (cyclophosphamide, doxorubicin, vincristine, and prednisolone) (R-CHOP)-like regimen regardless of the presence of MPM. In the 123 DLBCL patients with MPM, 103, 16, and 4 had one, two, and three other primary malignancies, respectively, before treatment for DLBCL. Stomach cancer was the most common, followed by colorectal cancer, lung cancer, prostate cancer, and breast cancer. Hematologic malignancies were less common than solid cancers as both past cancer or synchronous cancer (Supplementary Table S2).

We next investigated the prognostic impact and clinical characteristics of MPM in DLBCL. The median follow-up period was 899 days (range: 1 to 3609 days) for all 809 patients, and was significantly shorter in DLBCL patients with MPM compared to those without MPM (719 vs. 970.5 days, *P* = 0.015) (Supplementary Table S1). Both overall survival (OS) and progression-free survival (PFS) in patients with MPM were significantly shorter than in those without MPM (3-year OS: 56.2 vs. 74.6%, *P* < 0.001, median OS: both not reached; 3-year PFS: 49.3 vs. 64.2%, *P* < 0.01, median PFS: 907 vs. 2400 days, *P* < 0.01) (Fig. 1a, b). There were no significant differences in OS and PFS between patients with past cancer and synchronous cancer, and survival curves for OS and PFS in these groups were largely superimposed in our cohort (Supplementary Fig. S1). Given that the prognosis of patients with past cancer and synchronous cancer were statistically equivalent, these groups were combined into a single cohort of DLBCL patients with MPM for further investigation. As the result, MPM emerged as an independent risk factor for shorter OS (hazard ratio (HR) = 1.68; 95% confidence interval (CI) = 1.22–2.31, *P* < 0.001) and PFS (HR = 1.58; 95% CI = 1.19–2.09, *P* = 0.002), along with age, clinical stage, and IPI disease risk (Supplementary Table S3). We further investigated clinical characteristics and the prognostic impact of MPM in association with IPI-defined disease risk. More patient with MPM were over 60 years old compared with patients without MPM in the IPI-low risk group, but this age distribution did not differ significantly in the other risk groups. There were also no significant differences in gender, disease stage, or treatment regimen, regardless of the IPI-defined risk group (Table 1). However, the prognostic impacts of MPM were different among different IPI-defined risk groups, namely, the presence of MPM was significantly associated with shorter OS and PFS in IPI-low and IPI-high risk DLBCL, but not in other risk groups (Fig. 1c, d, e, f, Supplementary Fig. S2). Furthermore, the rate of death was significantly higher in patients with MPM than those without MPM in IPI-high risk group. In addition, the death by DLBCL tended to be more frequent in patients with MPM compared with those without MPM in the high risk group, although not statistically significant. Indeed, approximately 50% (16 of 30) IPI-high risk patients with MPM died due to progression of lymphoma (Table 1). In multivariate analysis, MPM was an independent poor prognostic factor in IPI-high risk DLBCL, but not in IPI-low risk patients (Supplementary Table S4).

**Fig. 1:**
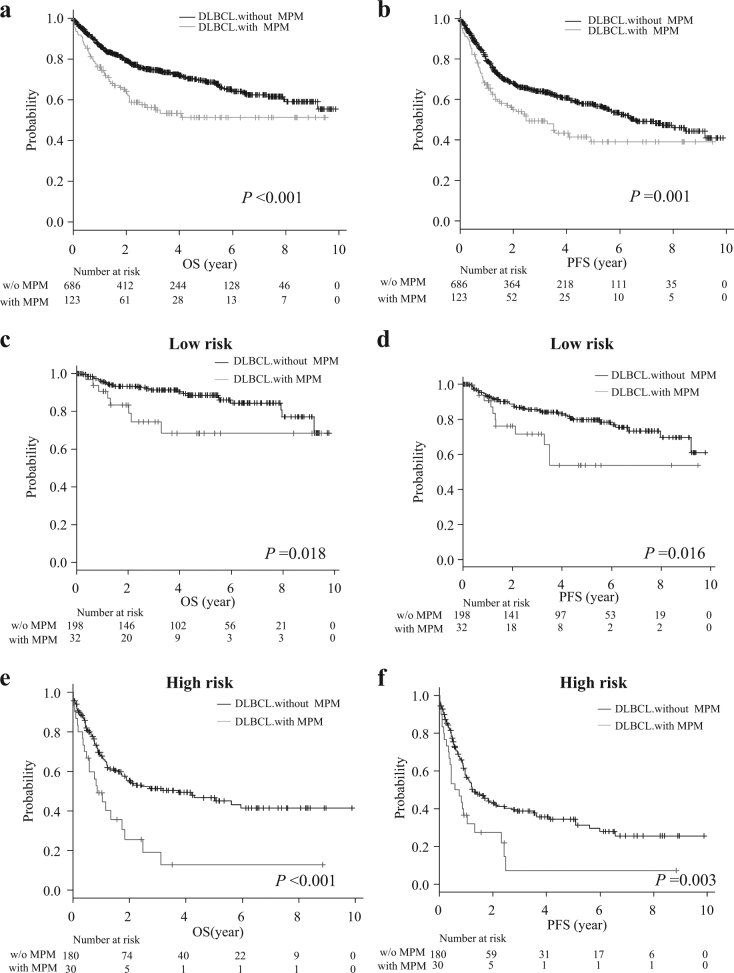
**a** Overall survival (OS) and **b** progression free survival (PFS) of DLBCL with and without (w/o) MPM. **c** OS and **d** PFS of IPI-defined low risk DLBCL patients with and without MPM. **e** OS and **f** PFS of IPI-defined high risk DLBCL patients with and without MPM

**Table 1:** Comparison of background, treatment and causes of death in DLBCL patients with MPM according to IPI risk groups

IPI	Low			Low-intermediate			High-intermediate			High		
MPM	+	−	*P*	+	−	*P*	+	−	*P*	+	−	*P*
N	32	198		31	142		30	166		30	180	
Age												
Median												
≤ 60	2	78	<0.001	4	30	0.3	6	33	0.99	0	19	0.06
≥ 61	30	120		27	112		24	133		30	161	
Gender												
Female	11	83	0.42	13	62	0.86	13	73	0.95	11	80	0.43
Male	21	115		18	80		17	93		19	100	
Stage												
I and II	32	193	0.36	26	69	< 0.001	6	29	0.74	2	6	0.16
III and IV	0	5		5	73		24	137		28	174	
Treatment												
R-CHOP like regimen	27	183	0.13	26	130	0.04	26	146	0.54	27	157	0.7
Intensive chemotherapy	0	5		3	1		2	8		2	6	
Treatment for PCNSL	1	2		0	3		1	3		0	2	
Others	2	2		1	2		1	2		0	5	
Unknown	2	6		1	6		0	8		1	10	
Cause of death			0.17			0.36			0.12			0.007
Number of death												
All death	8	24	0.051	10	28	0.13	12	52	0.35	22	85	0.008
Death by DLBCL	3	9	0.25	5	16	0.45	6	33	0.99	16	64	0.06
Death by other PCs	0	2	0.57	1	1	0.23	3	3	0.02	1	0	0.01
Death by other causative	5	13	0.08	4	11	0.36	3	16	0.95	5	21	0.44

The frequency of SPM should depend on the prognosis of the first malignancy. For instance, SPM should be less frequent in patients with cancers with a poor survival rate, such as pancreatic carcinoma, but more frequent in cancers with better prognosis, such as low grade thyroid cancer^1,10^. Due to the retrospective study design, we could not analyze the precise pathologic features of previous cancers, treatment efficacy for past cancer, or the detailed adverse events with chemotherapy for past cancer before DLBCL treatment, however, the types and frequencies of other cancers in our cohort of DLBCL patients with MPM, mostly reflected those in cancer surveillance research in Japan^11^. The type of past or synchronous malignancy did not differ significantly among patients according to the IPI risk (Supplementary Table S5). Stomach cancer was the most frequent in both past cancer and synchronous cancer cases, and approximately two-thirds (65.5%) of concomitant cancers were detected in upper gastrointestinal screening. It is also noteworthy that DLBCL, stomach cancer, and upper aerodigestive tract cancer occasionally share an etiology of exposure to chronic inflammation^12,13^. Although we were not able to address the underlying common etiology for synchronous cancers of DLBCL and stomach, and upper aerodigestive cancers, we found that 29 (3.5%) of 809 patients were diagnosed with DLBCL with synchronous cancer, indicating that careful systemic screening, including that of the upper aerodigestive tract is needed at the time of initial diagnosis of DLBCL.

While the choices of treatment regimen did not differ significantly between DLBCL patients with and without MPM, our multivariate analysis revealed that the negative prognostic impact of MPM was significant only in IPI-high risk DLBCL. Given that synchronous MPM including DLBCL is sometimes associated with an aggressive clinical course and a dismal prognosis^14,15^, we speculate that a paraneoplastic effect occasionally promotes disease progression and causes early death due to DLBCL, especially in IPI-high risk patients. In addition, it is also conceivable that a proportion of patients with MPM in our cohort harbored unverified mechanisms, such as inherited gene abnormalities associated with tumor development or immune surveillance, or environmental factors that underlie development of MPM and cause treatment-refractory disease.

In conclusion, approximately 15% of DLBCL patients had the history of MPM in our cohort. While our study did not provide the evidence for the need for chemotherapeutic modification because of the presence of MPM in DLBCL, careful attention is needed especially for IPI-high risk patients with MPM to improve their survival, as the cumulated death rate was higher in those patients, perhaps due to various patient-oriented and MPM-associated factors, including treatment failure for DLBCL.

## Electronic supplementary material

Supplemental table 1

Supplemental table 2

Supplemental table 3

Supplemental table 4

Supplemental table 5

Supplemental figure 1

Supplemental figure 2

Related Manuscript File (supplemental imformation)
