# Supplementary material for: Prognostic impact of a past or synchronous second cancer in diffuse large B cell lymphoma
Source: Blood Cancer J. 2018 Jan 25;8(1):1. doi: 10.1038/s41408-017-0043-6 (PMC5802597; doi:10.1038/s41408-017-0043-6)
Supplement: Supplementary file 1 — Supplemental table 1 [file 41408_2017_43_MOESM1_ESM.doc]

|  | DLBCL with MPM | | | | DLBCL without MPM | P-value  with MPM  vs. w/o MPM |
| --- | --- | --- | --- | --- | --- | --- |
|  | Total | with PC | with SC | P-value  PC vs. SC |  |  |
| No. of patients | 123 | 94 (11.6%) | 29 (3.6%) |  | 686 (84.8%) |  |
| Age | 75 | 75 (36-92) | 75 (52-88) | 0.843^a^ | 70 (19-94) | <0.001^a^ |
| Gender | M:F=75:48 | M:F=51:43 | M:F=24:5 | 0.006^b^ | M:F=388:298 | 0.017^b^ |
| Stage |  |  |  | 0.402^b^ |  | 0.239^b^ |
| I | 30 | 21 (22.3%) | 9 (31%) |  | 130 (19%) |  |
| II | 36 | 31 (33.0%) | 5 (17.2%) |  | 167 (24.3%) |  |
| III | 17 | 12 (12.8%) | 5 (17.2%) |  | 111 (16.2%) |  |
| IV | 40 | 30 (31.9%) | 10 (34.5%) |  | 278 (40.5%) |  |
| IPI |  |  |  | 0.450^b^ |  | 0.625^b^ |
| Low | 32 | 23 (24.5%) | 9 (31%) |  | 198 (28.9%) |  |
| Low-int. | 31 | 27 (28.7%) | 4 (13.8%) |  | 142 (20.7%) |  |
| High-int. | 30 | 22 (23.4%) | 8 (27.6%) |  | 166 (24.2%) |  |
| High | 30 | 22 (23.4%) | 8 (27.6%) |  | 180 (26.2%) |  |
| Treatment |  |  |  | 0.053^b^ |  | 0.042^b^ |
| R-CHOP-like regimen | 106 | 80 (85.1%) | 26 (89.7%) |  | 616 (89.8%) |  |
| Intensified chemotherapy | 7 | 6 (6.3%) | 1 (3.4%) |  | 20 (2.9%) |  |
| Chemotherapy for PCNSL | 2 | 0 (0%) | 2 (6.8%) |  | 9 (1.3%) |  |
| Others | 4 | 4 (4.3%) | 0 (0%) |  | 11 (1.6%) |  |
| No data | 4 | 4 (4.3%) | 0 (0%) |  | 30 (4.4%) |  |
| Median days of  follow-up | 719 (9-3444) | 734.5 (9-3444) | 652 (124-2748) | 0.343^a^ | 970.5 (1-3609) | 0.015^a^ |

**Supplementary Table S1.** **Comparisons of clinical characteristics between DLBCL patients with and without multiple primary malignancy (MPM).**
