# Supplementary material for: Prognostic impact of a past or synchronous second cancer in diffuse large B cell lymphoma
Source: Blood Cancer J. 2018 Jan 25;8(1):1. doi: 10.1038/s41408-017-0043-6 (PMC5802597; doi:10.1038/s41408-017-0043-6)
Supplement: Supplementary file 2 — Supplemental table 2 [file 41408_2017_43_MOESM2_ESM.doc]

**Supplementary Table S2.** **Type of past or synchronic malignancies in DLBCL patients with MPM.**

|  | **Total** | | | **Past cancer** | | | **Synchronous cancer** | | |
| --- | --- | --- | --- | --- | --- | --- | --- | --- | --- |
|  | total | Male | female | total | male | female | total | Male | female |
| **Number of patients** | 123 | 75 | 48 | 94 | 51 | 43 | 29 | 24 | 5 |
| **Type of other cancer** |  |  |  |  |  |  |  |  |  |
| Solid tumor |  |  |  |  |  |  |  |  |  |
| stomach | 36 | 27 | 9 | 20 | 14 | 6 | 16 | 13 | 3 |
| colorectal | 25 | 17 | 8 | 20 | 13 | 7 | 5 | 4 | 1 |
| lung | 10 | 9 | 1 | 8 | 7 | 1 | 2 | 2 |  |
| prostate | 15 | 15 |  | 11 | 11 |  | 4 | 4 |  |
| Breast | 15 | 1 | 14 | 14 |  | 14 | 1 | 1 |  |
| Uterus | 10 |  | 10 | 10 |  | 10 |  |  |  |
| Liver | 5 | 4 | 1 | 5 | 4 | 1 |  |  |  |
| urinary bladder | 7 | 6 | 1 | 4 | 3 | 1 | 3 | 3 |  |
| upper aerodigestive tract | 5 | 4 | 1 | 2 | 2 |  | 3 | 2 | 1 |
| Kidney | 2 | 1 | 1 | 2 | 1 | 1 |  |  |  |
| pancreas | 3 | 1 | 2 | 3 | 1 | 2 |  |  |  |
| Skin | 1 | 1 |  | 1 | 1 |  |  |  |  |
| gall bladder | 2 | 1 | 1 | 2 | 1 | 1 |  |  |  |
| thyroid | 1 |  | 1 | 1 |  | 1 |  |  |  |
| Langerhans cell histiocytosis | 1 | 1 |  |  |  |  | 1 | 1 |  |
|  |  |  |  |  |  |  |  |  |  |
| Hematologic malignancy |  |  |  |  |  |  |  |  |  |
| MDS | 2 | 1 | 1 | 1 |  | 1 | 1 | 1 |  |
| MALT Lymphoma | 3 | 1 | 2 | 2 | 1 | 1 | 1 |  | 1 |
| ET | 1 | 1 |  | 1 | 1 |  |  |  |  |
| ALL | 1 |  | 1 | 1 |  | 1 |  |  |  |
| HL | 1 | 1 |  | 1 | 1 |  |  |  |  |
| AITL | 1 |  | 1 | 1 |  | 1 |  |  |  |
