# Supplementary material for: Prognostic impact of a past or synchronous second cancer in diffuse large B cell lymphoma
Source: Blood Cancer J. 2018 Jan 25;8(1):1. doi: 10.1038/s41408-017-0043-6 (PMC5802597; doi:10.1038/s41408-017-0043-6)
Supplement: Supplementary file 3 — Supplemental table 3 [file 41408_2017_43_MOESM3_ESM.doc]

**Supplementary Table S3. Statistical analysis of factors associated with overall survival (OS) and progression free survival (PFS) in DLBCL patients with and without MPM.**

|  | OS | | | | | | | PFS | | | | | | |
| --- | --- | --- | --- | --- | --- | --- | --- | --- | --- | --- | --- | --- | --- | --- |
|  | Univariate HR | | |  | Multivariate HR | | | Univariate HR | | |  | Multivariate HR | | |
|  | HR | 95% CI | *P* |  | HR | 95%CI | *P* | HR | 95%CI | *P* |  | HR | 95%CI | *P* |
| Age | 1.04 | [1.03 - 1.06] | <0.001 |  | 1.04 | [1.03 - 1.06] | <0.001 | 1.03 | [1.02 - 1.04] | <0.001 |  | 1.03 | [1.02 - 1.05] | <0.001 |
| Sex |  |  |  |  |  |  |  |  |  |  |  |  |  |  |
| Female | 1 |  |  |  | - |  | - | 1 |  |  |  | 1 |  |  |
| Male | 1.27 | [0.98 - 1.65] | 0.067 |  | - |  | - | 1.32 | [1.06 - 1.65] | 0.013 |  | 1.4 | [1.11 - 1.76] | 0.004 |
| MPM |  |  |  |  |  |  |  |  |  |  |  |  |  |  |
| absent | 1 |  |  |  | 1 |  |  | 1 |  |  |  | 1 |  |  |
| present | 1.79 | [1.31 - 2.43] | <0.001 |  | 1.68 | [1.22 - 2.31] | <0.001 | 1.56 | [1.19 - 2.06] | 0.001 |  | 1.58 | [1.19 - 2.09] | 0.002 |
| Clinical stage |  |  |  |  |  |  |  |  |  |  |  |  |  |  |
| I and II | 1 |  |  |  | 1 |  |  | 1 |  |  |  | 1 |  |  |
| III and IV | 2.03 | [1.55 - 2.66] | <0.001 |  | 2.2 | [1.69 - 2.91] | <0.001 | 2.28 | [1.80 - 2.87] | <0.001 |  | 2.47 | [1.94 - 3.16] | <0.001 |
| IPI |  |  |  |  |  |  |  |  |  |  |  |  |  |  |
| Low and Low-Int. | 1 |  |  |  | 1 |  |  | 1 |  |  |  | 1 |  |  |
| High-int. and High | 3.06 | [2.35 - 4.06] | <0.001 |  | 2.85 | [2.15 - 3.77] | <0.001 | 2.75 | [2.19 - 3.46] | <0.001 |  | 1.48 | [1.41 - 2.71] | <0.001 |
| Treatment |  |  |  |  |  |  |  |  |  |  |  |  |  |  |
| R-CHOP like  regimen | 1 |  |  |  | 1 |  |  | 1 |  |  |  | 1 |  |  |
| Chemotherapy for  PCNSL | 0.44 | [0.06 - 3.17] | 0.42 |  | 0.51 | [0.07 - 3.66] | 0.5 | 0.6 | [0.15 - 2.42] | 0.48 |  | 0.68 | [0.17 - 2.72] | 0.582 |
| High intensive  chemotherapy | 3.2 | [1.84 - 5.48] | <0.001 |  | 3.27 | [1.88 - 5.08] | <0.001 | 2.82 | [1.70 - 4.69] | <0.001 |  | 2.85 | [1.71 - 4.76] | <0.001 |
| Others | 2.82 | [1.32 - 6.00] | 0.007 |  | 1.97 | [0.91 - 4.25] | <0.001 | 2.66 | [1.37 - 5.18] | <0.001 |  | 2.13 | [1.08 - 4.19] | 0.03 |
