# Supplementary material for: Prognostic impact of a past or synchronous second cancer in diffuse large B cell lymphoma
Source: Blood Cancer J. 2018 Jan 25;8(1):1. doi: 10.1038/s41408-017-0043-6 (PMC5802597; doi:10.1038/s41408-017-0043-6)
Supplement: Supplementary file 4 — Supplemental table 4 [file 41408_2017_43_MOESM4_ESM.doc]

**Supplementary Table S4. Multivariate analysis of prognostic factors, including MPM, according to IPI-defined disease risk.**

| IPI | Low risk | | | | | |  | High risk | | | | | |
| --- | --- | --- | --- | --- | --- | --- | --- | --- | --- | --- | --- | --- | --- |
|  | Univariate HR | | | Multivariate HR | | |  | Univariate HR | | | Multivariate HR | | |
|  | HR | 95% CI | *P* | HR | 95%CI | *P* |  | HR | 95%CI | *P* | HR | 95%CI | *P* |
| Age | 1.06 | [1.02- 1.10] | <0.01 | 1.06 | [1.02 – 1.10] | <0.01 |  | 1.04 | [1.02-1.07] | <0.001 | 1.04 | [1.02 - 1.07] | <0.001 |
| Sex |  |  |  |  |  |  |  |  |  |  |  |  |  |
| Female | 1 |  |  | - |  | - |  | 1 |  |  | - |  | - |
| Male | 1.79 | [0.84-3.83] | 0.13 | - |  | - |  | 1.14 | [0.78-1.67] | 0.5 | - |  | - |
| MPM |  |  |  |  |  |  |  |  |  |  |  |  |  |
| absent | 1 |  |  | 1 |  |  |  | 1 |  |  | 1 |  |  |
| present | 2.56 | [1.14-5.74] | 0.02 | 1.91 | [0.84 - 4.34] | 0.12 |  | 2.19 | [1.36- 3.52] | <0.01 | 1.97 | [1.22 – 3.16] | <0.01 |
| Clinical stage |  |  |  |  |  |  |  |  |  |  |  |  |  |
| I and II | Not evaluable |  |  | Not evaluable |  |  |  | 1 |  |  | - |  | - |
| III and IV |  |  |  |  |  |  |  | 1.17 | [0.31- 2.32] | 0.76 | - |  |  |

**-**

**Supplemental Table S1. Types of past cancer (PC) and synchronous cancer (SC) in DLBCL patients with MPM according to IPI-risk group**

| IPI | Low | | Low-intermediate | | High-intermediate | | High | |
| --- | --- | --- | --- | --- | --- | --- | --- | --- |
|  | PC | SC | PC | SC | PC | SC | PC | SC |
| N | 32 | 9 | 31 | 4 | 30 | 8 | 30 | 8 |
| Solid tumor |  |  |  |  |  |  |  |  |
| Stomach | 7 | 3 | 9 | 3 | 9 | 2 | 11 | 7 |
| Colon | 8 |  | 6 | 1 | 6 | 2 | 5 |  |
| Lung | 2 | 1 | 1 |  | 3 | 1 | 3 |  |
| Prostate | 3 | 1 | 3 |  | 4 | 2 | 5 | 1 |
| Breast | 5 | 1 | 5 |  | 1 |  | 4 |  |
| Uterus | 3 |  | 3 |  | 2 |  | 2 |  |
| Urinary bladder | 3 | 1 | 2 |  | 1 |  | 1 |  |
| Upper aerodigestive tract | 4 | 1 | 0 |  | 0 |  | 1 |  |
| Kidney | 1 |  | 0 |  | 1 |  | 0 |  |
| Pancreas | 2 |  | 1 |  | 0 |  | 0 |  |
|  |  |  |  |  |  |  |  |  |
| Hematologic cancer | 2 | 1 | 2 |  | 3 |  | 2 |  |
|  |  |  |  |  |  |  |  |  |
| Others | 2 |  | 2 |  | 4 | 1 | 3 |  |
