# Supplementary material for: Prognostic impact of a past or synchronous second cancer in diffuse large B cell lymphoma
Source: Blood Cancer J. 2018 Jan 25;8(1):1. doi: 10.1038/s41408-017-0043-6 (PMC5802597; doi:10.1038/s41408-017-0043-6)
Supplement: Supplementary file 5 — Supplemental table 5 [file 41408_2017_43_MOESM5_ESM.doc]

**Supplemental Table S5. Types of past cancer (PC) and synchronous cancer (SC) in DLBCL patients with MPM according to IPI-risk group**

| IPI | Low | | Low-intermediate | | High-intermediate | | High | |
| --- | --- | --- | --- | --- | --- | --- | --- | --- |
|  | PC | SC | PC | SC | PC | SC | PC | SC |
| N | 32 | 9 | 31 | 4 | 30 | 8 | 30 | 8 |
| Solid tumor |  |  |  |  |  |  |  |  |
| Stomach | 7 | 3 | 9 | 3 | 9 | 2 | 11 | 7 |
| Colon | 8 |  | 6 | 1 | 6 | 2 | 5 |  |
| Lung | 2 | 1 | 1 |  | 3 | 1 | 3 |  |
| Prostate | 3 | 1 | 3 |  | 4 | 2 | 5 | 1 |
| Breast | 5 | 1 | 5 |  | 1 |  | 4 |  |
| Uterus | 3 |  | 3 |  | 2 |  | 2 |  |
| Urinary bladder | 3 | 1 | 2 |  | 1 |  | 1 |  |
| Upper aerodigestive tract | 4 | 1 | 0 |  | 0 |  | 1 |  |
| Kidney | 1 |  | 0 |  | 1 |  | 0 |  |
| Pancreas | 2 |  | 1 |  | 0 |  | 0 |  |
|  |  |  |  |  |  |  |  |  |
| Hematologic cancer | 2 | 1 | 2 |  | 3 |  | 2 |  |
|  |  |  |  |  |  |  |  |  |
| Others | 2 |  | 2 |  | 4 | 1 | 3 |  |
