# Supplementary material for: Prognostic impact of a past or synchronous second cancer in diffuse large B cell lymphoma
Source: Blood Cancer J. 2018 Jan 25;8(1):1. doi: 10.1038/s41408-017-0043-6 (PMC5802597; doi:10.1038/s41408-017-0043-6)
Supplement: Supplementary file 6 — Supplemental figure 1 [file 41408_2017_43_MOESM6_ESM.pptx]

## Slide 1
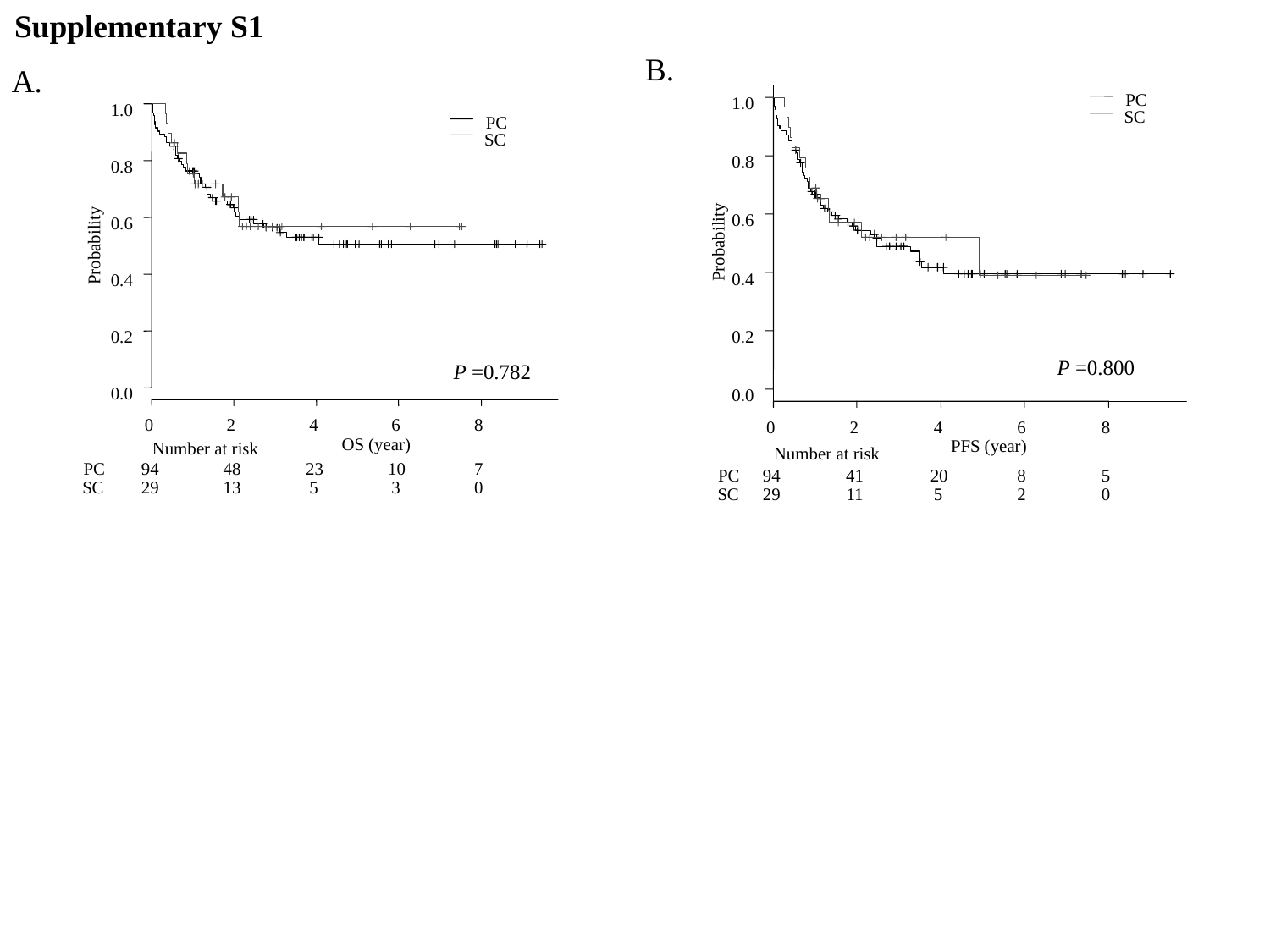

Supplementary S1
1.0
PC
SC
0.8
0.6
Probability
0.4
0.2
0.0
0
2
4
6
8
OS (year)
Number at risk
PC
94
48
23
10
7
SC
29
13
5
3
0
B.
A.
1.0
0.8
0.6
Probability
0.4
0.2
0.0
0
2
4
6
8
PFS (year)
Number at risk
PC
94
41
20
8
5
SC
29
11
5
2
0
PC
SC
P =0.800
P =0.782
