# Supplementary material for: Prognostic impact of a past or synchronous second cancer in diffuse large B cell lymphoma
Source: Blood Cancer J. 2018 Jan 25;8(1):1. doi: 10.1038/s41408-017-0043-6 (PMC5802597; doi:10.1038/s41408-017-0043-6)
Supplement: Supplementary file 7 — Supplemental figure 2 [file 41408_2017_43_MOESM7_ESM.pptx]

## Slide 1
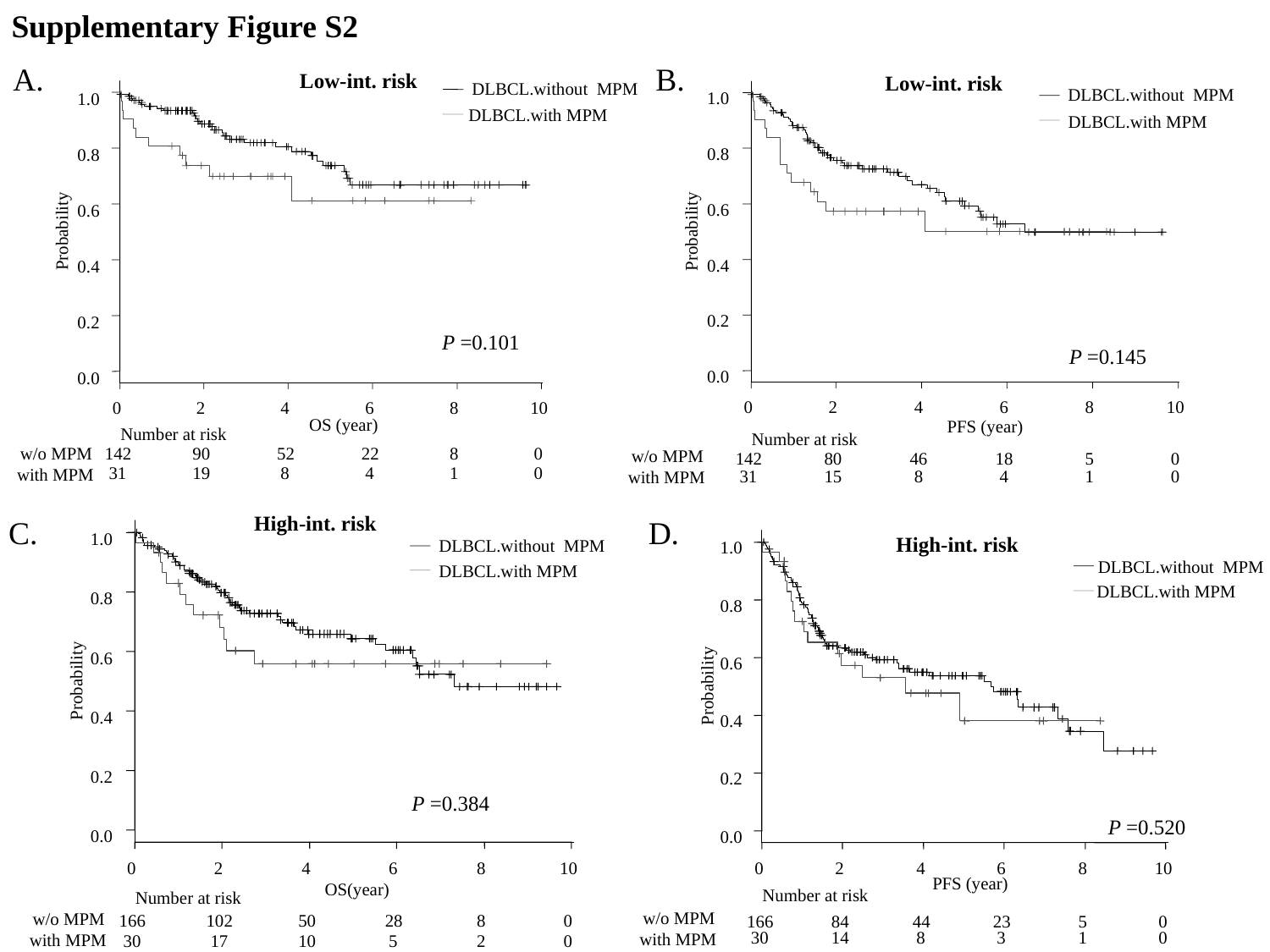

Supplementary Figure S2
Low-int. risk
1.0
0.8
0.6
Probability
0.4
0.2
0.0
0
2
4
6
8
10
PFS (year)
Number at risk
142
80
46
18
5
0
31
15
8
4
1
0
B.
DLBCL.without MPM
DLBCL.with MPM
P =0.145
w/o MPM
with MPM
A.
Low-int. risk
1.0
0.8
0.6
Probability
0.4
0.2
0.0
0
2
4
6
8
10
OS (year)
DLBCL.without MPM
DLBCL.with MPM
P =0.101
Number at risk
w/o MPM
142
90
52
22
8
0
with MPM
31
19
8
4
1
0
High-int. risk
1.0
0.8
0.6
Probability
0.4
0.2
0.0
0
2
4
6
8
10
OS(year)
Number at risk
166
102
50
28
8
0
30
17
10
5
2
0
C.
DLBCL.without MPM
DLBCL.with MPM
P =0.384
w/o MPM
with MPM
High-int. risk
1.0
0.8
0.6
Probability
0.4
0.2
0.0
0
2
4
6
8
10
PFS (year)
Number at risk
166
84
44
23
5
0
30
14
8
3
1
0
D.
DLBCL.without MPM
DLBCL.with MPM
P =0.520
w/o MPM
with MPM
