# Supplementary material for: Prognostic impact of a past or synchronous second cancer in diffuse large B cell lymphoma
Source: Blood Cancer J. 2018 Jan 25;8(1):1. doi: 10.1038/s41408-017-0043-6 (PMC5802597; doi:10.1038/s41408-017-0043-6)
Supplement: Supplementary file 8 — Related Manuscript File (supplemental imformation) [file 41408_2017_43_MOESM8_ESM.doc]

**Supplementary Information**

**Supplementary methods**

**Patients**

Data were collected for patient background, disease history, Ann Arbor disease stage, international prognostic index (IPI) [1], treatment procedure, response using Cheson’s revised criteria for malignant lymphoma [2], and overall survival (OS) and progression-free survival (PFS). Past cancer or synchronous cancer were confirmed from pathology findings or a review of medical records.

**Statistical analysis**

The characteristics of patients were analyzed by Chi-square test and Mann-Whitney U test. The cut-off date for analyses of PFS and OS was 31 January, 2016. PFS was defined as the period from the treatment start date until the date on which lymphoma progressed or the date of death from any cause. OS was calculated from the treatment start date until the date of final follow-up or of death from any cause. The median follow-up time was also calculated from the treatment start date. Survival curves for OS and PFS were estimated by the Kaplan-Meier method. Multivariate analyses were performed using Cox proportional-hazard models to estimate hazard ratios (HRs). *P* < 0.05 was considered significant. All analyses were performed using R3.1.2 (R Foundation for Statistical Computing, Vienna, Austria; http://www.R-project.org).

**Reference**

1. The International Non-Hodgkin’s Lymphoma Prognostic Factors Project. A predictive model for aggressive non-Hodgkin’s lymphoma. *N Engl J Med.* 1993; **329**: 987–94.

2. Cheson BD, Pfistner B, Juweid ME, Gascoyne RD, Specht L, Horning SJ, *et al*. Revised response criteria for malignant lymphoma. *J Clin Oncol.* 2007; **25**: 579-86.

**Supplementary Legends**

**Supplementaly Table S1.** **Comparisons of clinical characteristics between DLBCL patients with and without multiple primary malignancy (MPM).** ^a^Mann-Whitney U test, ^b^Chi-square test. M; male, F; female, IPI; International prognostic index, int; intermediate, PC; past cancer, SC; synchronous cancer, R-CHOP; rituximab plus cyclophosphamide, doxorubicin, vincristine and prednisolone. Most patients with advanced disease and approximately 80% with limited disease underwent 6-8 courses of this regimen, while a small number of patients with limited disease received 3 cycles of R-CHOP plus radiation or surgery as first-line therapy. A few patients with advanced disease were treated with high-dose chemotherapy supported by autologous hematopoietic stem cell transplantation as first-line treatment. Patients with primary central nervous system lymphoma received high-dose methotrexate therapy. Other therapies included more palliative approaches, such as radiotherapy, rituximab monotherapy, or low-dose R-GDP (rituximab, gemcitabine, dexamethasone, and cisplatin).

**Supplementary Table S2.** **Type of past or synchronic malignancies in DLBCL patients with MPM.** MDS, myelodysplastic syndrome; CML, chronic myelogenous leukemia; PMF, primary myelofibrosis; ET, essential thrombocythemia; ALL, acute lymphoblastic leukemia; HL, Hodgkin lymphoma; PTCL, NOS, peripheral T cell lymphoma, not otherwise specified; AITL, angioimmunoblastic T cell lymphoma.

**Supplementary Table S3.** **Statistical analysis of factors associated with overall survival (OS) and progression free survival (PFS) in DLBCL patients with and without MPM.** HR; Hazard ratio.

**Supplementary Table S4. Multivariate analysis of prognostic factors, including MPM, according to IPI-defined disease risk.**

**Supplemental Table S5. Types of past cancer (PC) and synchronous cancer (SC) in DLBCL patients with MPM according to IPI-risk group**

**Supplementary Figure S1. Long-term treatment outcomes of diffuse large B cell lymphoma (DLBCL) patients with and without MPM.** (A) Overall survival (OS) and (B) progression free survival (PFS) of with past cancer (PC) and synchronous cancer (SC).

**Supplementary Figure S2. Overal survival (OS) and Progression-free survival (PFS) of DLBCL patients with and without MPM according to IPI-defined disease risk.** (A) Low risk, (B) low-intermediate (Low-int) risk, (C) high-intermediate (High-int) risk, and (D) high risk.

(**A**) OS and (**B**) PFS of IPI-defined low-intermediate (Low-int) risk DLBCL patients with and without MPM. (C) OS and (D) PFS of IPI-defined high-intermediate (High-int) risk DLBCL patients with and without MPM.
